# Effectiveness of a virtual reality-based cognitive training program on memory, executive functioning, and processing speed in individuals with substance use disorders: a quasi-experimental study

**DOI:** 10.3389/fnbeh.2025.1653783

**Published:** 2025-09-19

**Authors:** Tânia Caetano, Maria Salomé Pinho, Eduardo Ramadas, Filipa Freire-Santos, Maria dos Anjos Dixe

**Affiliations:** ^1^Faculty of Psychology and Educational Sciences of University of Coimbra, Center for Research in Neuropsychology and Cognitive and Behavioural Intervention (CINEICC), University of Coimbra, Coimbra, Portugal; ^2^Center for Innovative Care and Health Technology (ciTechcare), Polytechnic of Leiria, Leiria, Portugal; ^3^Research and Innovation Department, VillaRamadas International Treatment Centre, Leiria, Portugal

**Keywords:** substance use disorders, cognitive training, virtual reality, VRrainSUD-VR, effectiveness, memory, executive functions

## Abstract

**Background:**

Cognitive deficits are frequently observed in individuals with Substance use disorders (SUD) and have been linked to poorer treatment outcomes and a heightened risk of relapse. We aimed to study the effectiveness of a 6-week virtual reality-based cognitive training program (VRainSUD-VR) on neuropsychological outcomes, specifically memory, executive functioning, and processing speed, as well as on treatment dropout rates in individuals with SUD. We hypothesized that adding VRainSUD-VR to treatment as usual (TAU) would lead to greater cognitive improvements compared to TAU alone. As a secondary hypothesis, we expected VRainSUD-VR to reduce false memories relative to TAU.

**Methods:**

A non-randomized design with a control group, pre- and post-test assessments, and convenience sampling was employed. Patients (*N* = 47) were assigned to either the control group (CG), which received TAU (*n* = 22), or the experimental group (EG), which received VRainSUD-VR in addition to TAU (*n* = 25). Cognitive and treatment outcomes (e.g., dropout rates) were assessed at pre- and post-test.

**Results:**

Statistically significant time × group interactions were found for overall executive functioning [*F*_(1, 75)_ = 20.05, *p* < 0.001] and global memory [*F*_(1, 75)_ = 36.42, *p* < 0.001], indicating the effectiveness of VRainSUD-VR. No significant time × group interactions were found for most processing speed outcomes (*p* > 0.05).

**Conclusion:**

VRainSUD-VR could be integrated into residential programs to improve general executive functioning, perceptual reasoning, and working memory, including visual working memory, as well as different aspects of global memory, such as visual, auditory, immediate, and delayed recall. Future research should explore the long-term effects of this intervention and consider additional potential mediating factors to gain a deeper understanding of the mechanisms underlying its effectiveness.

## 1 Introduction

Substance use disorders (SUD) are chronic, relapsing conditions characterized by cognitive, behavioral, and physiological symptoms, in which individuals persist in substance use despite harmful consequences (American Psychiatric Association, 2013). In recent years, the growing burden of these disorders has highlighted the urgent need for the development of effective interventions (Chisholm et al., 2006; Peacock et al., 2018; Shen et al., 2023).

Cognitive impairment is a common feature across several neuropsychiatric disorders, including SUD (Melugin et al., 2021). The addiction cycle is reinforced by impaired top-down cognitive control, which disrupts goal-driven behaviors and negatively impacts treatment outcomes (Cunha et al., 2010; Domínguez-Salas et al., 2016). Therefore, beyond compromising everyday functioning and wellbeing, cognitive deficits pose a known risk factor for treatment dropout and relapses (Brandon et al., 2007; Brorson et al., 2013). Neuroimaging studies reveal underlying structural and functional changes resulting from chronic substance use, regardless of the drug of choice (Hill-Bowen et al., 2022; Yan et al., 2023), and even short periods of abstinence from substance use aid in recovering brain function (Parvaz et al., 2022). Current gold-standard treatment may not fully address the neurocognitive factors sustaining substance-related addictive behaviors, making it difficult for individuals to benefit from such treatments. However, the complementary administration of neurocognitive interventions may further enhance treatment outcomes and support long-term recovery (Nardo et al., 2022; Sofuoglu et al., 2013).

Several cognitive functions, including memory, executive functions, and processing speed, are significantly impaired in chronic users of various substances (Potvin et al., 2014; Verdejo-Garcia et al., 2019). Additionally, substances such as alcohol and cannabis can increase susceptibility to false memories, which are frequently recalled with strong conviction (Kloft et al., 2021; Pardilla-Delgado and Payne, 2017). The confidence in these memories is related to the prefrontal cortex's ability to create mental representations resembling actual learned experiences (Kim et al., 2022). Consequently, provoked confabulations are associated with deficits in memory and executive function (Abadie and Camos, 2019; Cunningham et al., 1997).

Cognitive training programs are interventions designed to improve specific cognitive functions through the repeated practice of structured tasks. For individuals suffering from SUD, cognitive training has the potential to enhance cognitive functions and influence clinical outcomes such as treatment dropout rates (Anderson et al., 2023; Caetano et al., 2021). Cognitive deficits, such as executive dysfunction, may serve as a fundamental psychopathological mechanism driving compulsive substance use, while improving these functions may enhance self-regulation and lower relapse risk (Brooks et al., 2017; Giancola and Tarter, 1999; Khemiri et al., 2019; Marceau et al., 2017). Therefore, targeting cognitive functions through cognitive training could improve overall treatment outcomes. Most traditional cognitive training programs rely on paper-and-pencil exercises, which may lack interactivity, motivation, and engagement for patients (Pazoki et al., 2024; Zuo, 2022).

Virtual reality (VR) enables the creation of immersive, interactive 3D computer-generated environments that replace real-world sensory input, providing precise control for therapeutic strategies and ensuring consistent, repeatable treatment (Freeman et al., 2017). Despite growing interest in the use of VR in clinical settings, its application in the context of SUD remains relatively underexplored. Systematic reviews have shown that existing studies have primarily focused on cue exposure and craving induction (Langener et al., 2021; Tsamitros et al., 2021), with limited evidence for effects on cognitive outcomes. According to Wankhede et al. (2025), VR contributes significantly to neurobiological changes, especially in sensory feedback, neuronal connectivity, motor learning, and cognitive functions. The immersive nature of VR environments may stimulate neuroplasticity and enhance hippocampal function (Clemenson and Stark, 2015), reinforcing its role as a tool for cognitive training.

VR-based cognitive training has gained immense interest in cognitive rehabilitation, primarily for age-related cognitive decline (Bauer and Andringa, 2020) and neurological conditions such as mild cognitive impairment and dementia (Papaioannou et al., 2022; Wu et al., 2020; Zhong et al., 2021). Prior VR-based cognitive training studies in SUD have focused exclusively on alcohol use disorder populations (Gamito et al., 2021; Pennington et al., 2022), with tasks emphasizing real-life activities rather than domain-specific cognitive training (Man, 2020).

In the present study, we aimed to evaluate the effectiveness of a 6-week VR-based cognitive training platform (VRainSUD-VR) when added to treatment as usual (TAU) compared to TAU alone, focusing on neuropsychological and treatment outcomes in patients with SUD.

As **primary hypotheses**, we hypothesized that patients receiving VRainSUD-VR plus TAU would demonstrate significantly improved cognitive performance in memory, executive functions, and processing speed compared to those receiving TAU alone. As **secondary hypotheses**, we hypothesized that participants undergoing the experimental intervention would have fewer false memories compared to the control group, especially among those showing significant improvements in cognitive functioning and memory. Additionally, we expect a lower dropout rate in the experimental group.

## 2 Methods

This quasi-experimental study used a non-randomized design with cluster allocation, a control group, pre- and post-test assessments, and convenience sampling of Dutch patients from a treatment center in Leiria, Portugal. Participants were assigned to either the experimental group [EG; VRainSUD-VR plus treatment as usual (TAU)] or the control group (CG; TAU only) based on the treatment unit they attended. The two clinics belonged to the same treatment center, specialized in the treatment of SUD, and were pre-designated to serve as either the EG or the CG. Patient assignment to each clinic was determined solely by the center's administrative procedures and was entirely independent of the research team. It is important to note that the allocated clinics implement an identical TAU program. This study received approval from the Ethics Committee of the Faculty of Psychology and Social Sciences at the University of Coimbra. Ethical considerations followed the principles outlined in the Declaration of Helsinki. We obtained written informed consent from all participants.

### 2.1 Participants

Participants were patients admitted to a residential SUD treatment program who (1) were at least 18 years old, (2) had a diagnosis of SUD according to DSM-5 criteria, (3) exhibited severe substance use according to either the Alcohol Use Disorders Identification Test (AUDIT; Babor et al., 2001) or the Drug Use Disorders Identification Test (DUDIT; Berman et al., 2003), and (4) were fluent in English. Exclusion criteria included concurrent gaming addiction, neurological conditions, and clinically significant symptoms of depression according to the Center for Epidemiologic Studies Depression Scale Revised (CESD-R-20; Radloff, 1977).

The inclusion and exclusion of participants were primarily based on clinical assessments carried out by the psychiatric team at the center where the data were collected. These assessments adhered to the standard procedures of the centers and were based on the DSM-5 diagnostic criteria. They involved clinical observation and semi-structured interviews conducted following the institution's procedures, supplemented by measures such as the CES-D, AUDIT, and DUDIT, to enhance the detection of clinically relevant symptoms.

**Figure 1:**
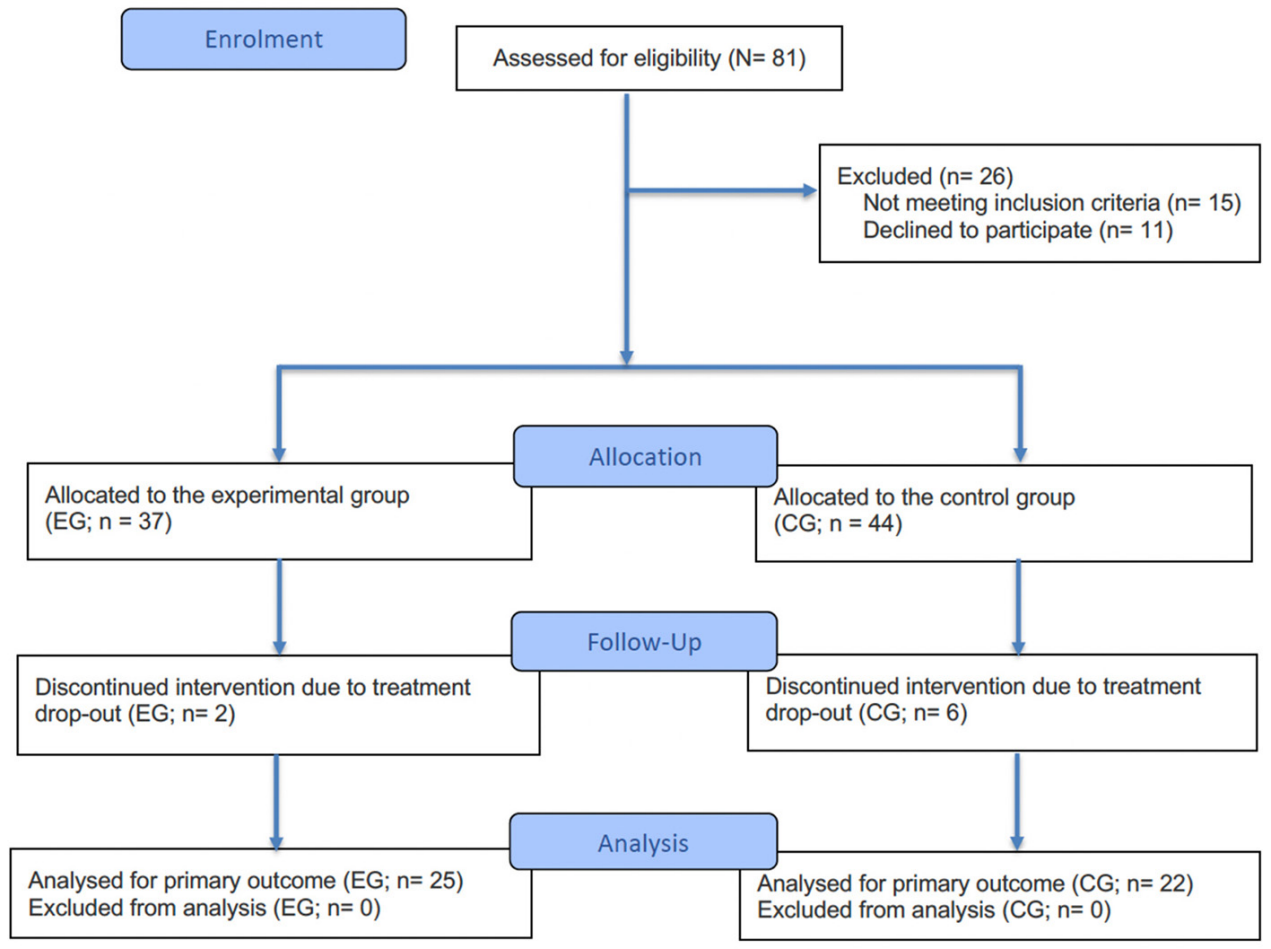
Consort diagram. Follow-up refers to the 6-week intervention period between the pre- and post-intervention assessments.

Recruitment occurred from January 2022 to January 2025. Study staff typically informed participants about the study during their initial days of treatment. As illustrated in the CONSORT diagram (Figure 1; Hopewell et al., 2025), out of the 81 patients assessed for eligibility, a total of 26 patients were excluded at enrolment (EG, *n* = 10; CG, *n* = 16) due to not meeting the inclusion criteria (EG, *n* = 5; CG, *n* = 10) or declining participation (EG, *n* = 5; CG, *n* = 6). During the intervention period, eight participants dropped out of treatment (EG, *n* = 2; CG, *n* = 6) and did not complete the 6-week follow-up. In total, 47 participants (EG, *n* = 25; CG, *n* = 22) met the study criteria, provided written informed consent, and completed pre- and post-intervention assessments.

The VRainSUD-VR program was implemented by psychologists from the treatment center, who received training in its use. The evaluation (pre- and post-test) was not carried out by the same professionals.

### 2.2 Sample size

For reference and methodological comparison, the G^*^Power 3.1 software (Faul et al., 2009) was used to obtain a theoretical estimate of the ideal sample size for a desired statistical power of 0.80, based on Cohen's (1988) effect size specification. We used repeated measures ANOVA with a group × time interaction, a significance level of α = 0.05, and an effect size of *f* = 0.403 derived from the average effect sizes reported in three studies involving cognitive training interventions in individuals with a history of substance abuse (Khemiri et al., 2019; Rezapour et al., 2019; Rupp et al., 2012). The calculation indicated an ideal sample of 52 participants in total (26 per group).

Since the sample in this effectiveness study comprises a total of 47 participants (*n* = 22 in the control group and *n* = 25 in the experimental group), a *post-hoc* statistical power calculation was performed using the same parameters and the effective sample size of the study, yielding a statistical power of 0.77. Although slightly below the conventional standard of 0.80, it demonstrates adequate statistical power to identify effects of moderate to high magnitude (Maxwell and Delaney, 2004).

Cohen (1992) suggests that values below 0.80, even if only slightly lower, should be viewed with caution because they might hinder identifying smaller true effects, thereby increasing the chance of type II errors.

### 2.3 Residential treatment program

All patients included in this study received the usual treatment (TAU) provided by the treatment center. The residential treatment program offers knowledge, techniques, and skills to better cope with addictive disorders and prevent relapse (e.g., individual and group therapy; psychoeducation; acceptance- and mindfulness-oriented techniques; relapse prevention; cognitive-behavioral therapy). All allocated clinics for this study follow the same TAU program, with standardized routines and procedures. TAU includes daily 1-h individual therapy sessions integrating components of cognitive-behavioral therapy, humanistic approaches, and other client-centered models. Additionally, four daily group therapy sessions are offered, including psychoeducational interventions, relapse prevention groups, and psychosocial support. Patients also received weekly medical and psychiatric consultations. The frequency, duration, and content of TAU followed the center's institutional protocol, ensuring homogeneity across clinics. Although there were minor differences in staff composition, all professionals received structured training and ongoing supervision from the institution. In the residential treatment program, there is no intervention focused on cognitive functioning.

### 2.4 Study groups

#### 2.4.1 Experimental group

Participants assigned to the experimental group (EG) received the VRainSUD-VR program in addition to their usual treatment. VRainSUD-VR was scheduled three times a week, with each session lasting 30 min, over a total of 6 weeks (see Caetano et al., 2025).

#### 2.4.2 Control group

In the control group (CG), participants only received the standard residential treatment for SUD. They completed all the same baseline and follow-up assessments as the patients in the experimental group (EG).

### 2.5 Assessments

#### 2.5.1 CESD-R-20

The CESD-20-R is a revised version of Radloff's original CES-D (Radloff, 1977). The revised version was adapted by Eaton based on the DSM-IV diagnostic criteria for major depressive episodes. It is a 20-item self-assessment scale used to support the assessment of depressive symptoms (Eaton et al., 2004; Van Dam and Earleywine, 2011). The scale score ranges from 0 to 80, with higher scores indicating more depressive symptoms. It demonstrates strong psychometric properties, including high internal consistency (Van Dam and Earleywine, 2011).

#### 2.5.2 DUDIT

The DUDIT is a screening test used as a measure for inclusion criteria to assess problematic use of illegal substances. DUDIT consists of 11 items that evaluate frequency, dependence symptoms, and drug-related consequences, with a clinical threshold indicating problematic drug use of ≥6 for men and ≥2 for women (Berman et al., 2003). This screening test demonstrates high internal consistency (Hildebrand, 2015).

#### 2.5.3 AUDIT

The AUDIT is a validated screening test used as a measure of inclusion criteria to assess problematic alcohol use. AUDIT is a 10-item screening tool developed to evaluate alcohol consumption, dependence, and harmful consequences, with a clinical threshold indicating problematic drug use of ≥8 for men and ≥6 for women (Babor et al., 2001).

#### 2.5.4 Sociodemographic questionnaire

The sociodemographic data were collected at baseline to assess age, sex, marital status, education level, clinical diagnosis, frequency and duration of drug use, age at first drug use, history of overdose, and drug of choice.

#### Trail making test A and B (TMT-A and -B)

2.5.5 Trail making test A and B (TMT-A and -B)]2.5.5 Trail making test A and B (TMT-A and -B)

*Trail Making Test A and B (TMT-A and -B; Reitan*, *1958**)* is divided into two parts: TMT-A requires connecting numbers in ascending order, whereas TMT-B involves alternating between numbers and letters in sequential order. TMT-A measures processing speed, whereas TMT-B assesses cognitive flexibility. Longer completion times indicate poorer performance. TMT has demonstrated strong reliability as a measure of executive functions (Reitan and Wolfson, 1993).

#### Frontal assessment battery (FAB)

2.5.6 Frontal assessment battery (FAB)]2.5.6 Frontal assessment battery (FAB)

The FAB is a reliable and valid tool that comprises six tasks designed to assess cognitive abilities related to frontal lobe function, particularly executive functions: lexical fluency, conflicting instructions, go–no–go, similarities, motor series, and prehension behavior (Dubois et al., 2000).

#### Stroop test

2.5.7 Stroop test]2.5.7 Stroop test

The computerized version of the Stroop Color-Word Task (SCWT) developed using the open-source Psychology Experiment Building Language (PEBL Project, 2018) was used to assess cognitive flexibility and inhibitory control (Cunha et al., 2010; Stroop, 1935; Mueller and Piper, 2014). The task included three blocks (color-word naming, black color-word naming, and colored patches naming), each with word-naming and color-naming conditions. Stimuli consisted of congruent, incongruent, and neutral trials using the words “red,” “green,” “blue,” and “yellow.” After 50 practice trials, participants completed 900 experimental trials (150 per set). Stimuli were presented individually in lowercase, and participants responded by pressing the corresponding key as quickly and accurately as possible.

#### Wechsler Adult Intelligence Scale—Fourth Edition (WAIS-IV

2.5.8 Wechsler Adult Intelligence Scale—Fourth Edition (WAIS-IV)]2.5.8 Wechsler Adult Intelligence Scale—Fourth Edition (WAIS-IV)

The WAIS-IV is a well-known instrument that measures overall cognitive functioning, memory, attention, and problem-solving skills (Wechsler, 2008). It includes several subtests: arithmetic, digit span, letter-number sequencing, picture completion, coding, symbol search, and visual puzzles. Individuals with SUD demonstrate greater cognitive impairment on WAIS measures of verbal comprehension and processing speed (Sullivan et al., 2023).

#### Wechsler Memory Scale—Fourth Edition (WMS-IV)

2.5.9 Wechsler Memory Scale—Fourth Edition (WMS-IV)]2.5.9 Wechsler Memory Scale—Fourth Edition (WMS-IV)

The WMS-IV is a standardized test that assesses memory functions and consists of six subtests: spatial addition, symbol span, logical memory (I & II), verbal paired associates (I & II), design (I & II), and visual reproduction (I & II) (Wechsler, 2009). The WMS-IV demonstrates satisfactory reliability, with coefficients ranging from 0.93 (for the visual working memory index) to 0.96 (for the visual memory index), along with solid construct and concurrent validity (Drozdick et al., 2011).

#### Wisconsin Card Sorting Test (WCST)—Computerized Berg’s card-sorting test version

2.5.10 Wisconsin Card Sorting Test (WCST)—Computerized Berg's card-sorting test version]2.5.10 Wisconsin Card Sorting Test (WCST)—Computerized Berg's card-sorting test version

The WCST measures executive functions by requiring participants to match 128 response cards to one of four key cards based on perceptual rules (Grant and Berg, 1948; Mueller and Piper, 2014; PEBL Project, 2018). Once the correct rule is learned, it must be applied across changing stimuli. After 10 correct matches, the rule shifts without warning, testing cognitive flexibility. Indexes of perseverative responses and perseverative errors are used to assess cognitive flexibility (Miles et al., 2021).

#### Dalla Barba confabulation battery (CB)

2.5.11 Dalla Barba confabulation battery (CB)]2.5.11 Dalla Barba confabulation battery (CB)

CB allows for the quantification and qualification of different types of provoked confabulations through questions about orientation in time and place, episodic memory, personal semantic memory, and items designed to elicit a “Don't Know” response (Dalla Barba, 1993; adapted the United Kingdom version by Kopelman et al., 1997). Only the general semantic and orientation sections of the confabulation battery were administered. Participants are considered confabulators if they produce 15% or more confabulations in the episodic memory section questions (Dalla Barba et al., 2019; Serra et al., 2014).

#### Deese-Roediger-McDermott paradigm (DRM paradigm)

2.5.12 Deese-Roediger-McDermott paradigm (DRM paradigm)]2.5.12 Deese-Roediger-McDermott paradigm (DRM paradigm)

DRM is a robust and reliable measure of false recognition or false recall (Deese, 1959; Roediger and McDermott, 1995). In this paradigm, lists of associatively related words are presented during encoding (e.g., mad, fear, hate, rage, and temper), with one highly related theme word (the “critical lure”: anger) not being presented. Critical lures are often incorrectly recalled or recognized as having been previously shown, which leads to the creation of false memories (Brainerd et al., 2008).

### 2.6 Data analysis

The data analyses were conducted using IBM SPSS Statistics, version 29 (IBM Corp, 2022). Descriptive statistics were used to assess demographic data. The chi-square test was used to compare categorical variables, whereas Student's *t*-test was used to evaluate sociodemographic differences between the experimental and the control groups for continuous variables.

The Generalized Linear Model with Mixed Effects (GLMM) was used to evaluate the effectiveness of the cognitive training program. This model allowed for the assessment of the intervention by collecting data at two evaluation moments (pre- and post-test), incorporating fixed effects (time-group interaction) along with random effects (considering the individual differences of the participants). GLMM with variance components was used, and the model was adjusted for the following covariates: age, clinical diagnosis, drug use time, marital status, and substance type. Simple contrasts with *post-hoc* comparisons were implemented, using the Sequential Bonferroni adjustment for multiple comparisons to control type I errors.

We also used a linear regression model with an interaction term between executive functioning and memory, as well as the group, to verify whether improvements in executive functioning and/or memory were associated with a decrease in false memories in the experimental group.

Correlation analyses between groups were also performed using Pearson or Spearman tests, depending on the data distribution, to explore the relationship between neuropsychological variables and specific sociodemographic variables of the study, including age and time of use. Comparisons between groups were also conducted using ANOVA or Kruskal-Wallis tests based on the assumptions of normality to assess differences in neuropsychological variables as a function of education and drug of choice.

Statistical significance was set at *p* < 0.05, with suitable adjustments applied for multiple comparisons when necessary.

## 3 Results

### 3.1 Sample description

Table 1 summarizes the demographic characteristics for the entire sample, as well as for participants assigned to each group separately. The two groups did not differ on any demographic variables (all *p* > 0.05). Participants were in their mid-30s (*M* = 35.83, *SD* = 9.15, range 19–62 years), with the majority being single (83%) and having at least a secondary or higher education (93.7%). The most prevalent clinical diagnoses were Alcohol Use Disorder (29.8%), Cocaine Use Disorder (21.3%), and Cannabis Use Disorder (10.6%), with many individuals experiencing polysubstance use (27.7%). Accordingly, the most common drugs of choice were alcohol (31.9%), cocaine (36.2%), and cannabis (14.9%). Over half of the participants (53.2%) reported consuming substances more than once daily, with the average age of first use at 17.28 years and an average duration of use of 11.15 years. The majority (80.9%) reported never experiencing an overdose. Individuals diagnosed with depression were identified before entering treatment. However, to complement the psychiatric team's clinical assessment of depression at admission, the CESD-20-R was administered, which assessed the frequency of depression symptoms based on the Diagnostic and Statistical Manual of Mental Disorders (DSM-5; American Psychiatric Association, 2013). The average AUDIT and DUDIT scores were both above 20, indicating an elevated risk for alcohol use disorder and drug use disorder, respectively.

**Table 1:** Sociodemographic data of the participants.

	**Total (*n* = 47) Mean ±SD/*N* (%)**	**Experimental group (*n* = 25) Mean ±SD/*N* (%)**	**Control group (*n* = 22) Mean ±SD/*N* (%)**	***t*/*X*^2^**	***p***
Age	35.83 ± 9.15	34.88 ± 8.66	36.91 ± 9.77	0.755	0.454^a^
**Sex**				0.503	0.478^b^
Male	34 (72.3 %)	17 (68.0%)	17 (77.3%)		
Female	13 (27.7%)	8 (32.0%)	5 (22.7%)		
**Marital status**				5.472	0.065^b^
Single	39 (83%)	22 (88.0%)	17 (77.3%)		
Married	4 (8.5%)	3 (12.0%)	1 (4.5%)		
Divorced	4 (8.5%)	0 (0.0%)	4 (18.2%)		
**Education level**				5.689	0.128^b^
Elementary school	3 (6.4%)	0 (0.0%)	3 (13.6%)		
Secondary education	35 (74.5%)	20 (80.0%)	15 (68.2%)		
Bachelor's degree	7 (14.9%)	3 (12.0%)	4 (18.2%)		
Master's degree	2 (4.3%)	2 (8.0%)	0 (0.0%)		
**Clinical diagnosis**				5.183	0.394^b^
Polysubstance use	13 (27.7%)	6 (24.0%)	7 (31.8%)		
Cannabis use disorder	5 (10.6 %)	4 (16.0%)	1 (4.5%)		
Alcohol use disorder	14 (29.8%)	9 (36.0%)	5 (22.7%)		
Cocaine use disorder	10 (21.3%)	5 (20.0%)	5 (22.7%)		
Opioid use disorder	2 (4.3%)	0 (0.0%)	2 (9.1%)		
Other psychoactive substance use disorder	3 (6.4%)	1 (4.0%)	2 (9.1%)		
**Frequency of drug use**				4.587	0.332^b^
Once a month	1 (2.1%)	0 (0.0%)	1 (4.5%)		
Two to four times a month	1 (2.1%)	0 (0.0%)	1 (4.5%)		
Two to three times a week	5 (10.6%)	3 (12.0%)	2 (9.1%)		
More than four times a week	15 (31.9%)	6 (24.0%)	9 (40.9%)		
More than once a day	25 (53.2%)	16 (64.0%)	9 (40.9%)		
Time use of drugs	11.15 ± 7.64	9.96 ± 7.12	12.50 ± 8.13	1.142	0.260^a^
Age at first drug use	17.28 ± 4.84	17.40 ± 5.74	17.14 ± 3.71	−0.184	0.855^a^
**Overdose**				0.812	0.368^b^
Yes	9 (19.1%)	6 (24.0%)	3 (13.6%)		
No	38 (80.9%)	19 (76%)	19 (86.4%)		
**Drug of choice**				4.772	0.573^b^
Alcohol	15 (31.9%)	9 (36.0%)	6 (27.3%)		
Crack	2 (4.3%)	1 (4.0%)	1 (4.5%)		
Cannabis	7 (14.9%)	5 (20.0%)	2 (9.1%)		
Oxycodone	1 (2.1%)	0 (0.0%)	1 (4.5%)		
Cocaine	17 (36.2%)	9 (36.0%)	8 (36.4%)		
Methamphetamine	4 (8.5%)	1 (4.0%)	3 (13.6%)		
Ritalin (methylphenidate)	1 (2.1%)	0 (0.0%)	1 (4.5%)		
CESD-R-20	11.62 ± 4.08	10.88 ± 4.55	12.45 ± 3.39	1.330	0.190^a^
AUDIT	22.53 ± 13.90	24.28 ± 13.01	20.55 ± 14.91	−0.918	0.364^a^
DUDIT	29.34 ± 15.74	28.56 ± 17.49	30.23 ± 13.84	0.364	0.717^a^

CESD-R-20, The Center for Epidemiologic Studies Depression Scale—Revised; AUDIT, Alcohol Use Disorders Identification Test; DUDIT, Drug Use Disorders Identification Test. ^a^Independent t-test. ^b^Chi-square test.

Regarding false memories, no participant in either group can be classified as a confabulator, as all produced fewer than 40% confabulations in questions related to episodic memory.

Concerning drop-out rates (EG, *n* = 25%; CG, *n* = 75%), no statistically significant differences were found between groups [χ^2^_(1)_ = 2.174, *p* = 0.140].

While adherence and potential adverse effects related to VR were not formally measured, no adverse effects were informally reported by participants, and all completed the full number of sessions (100% adherence).

### 3.2 Primary outcomes

#### 3.2.1 Executive functioning

**Figure 2:**
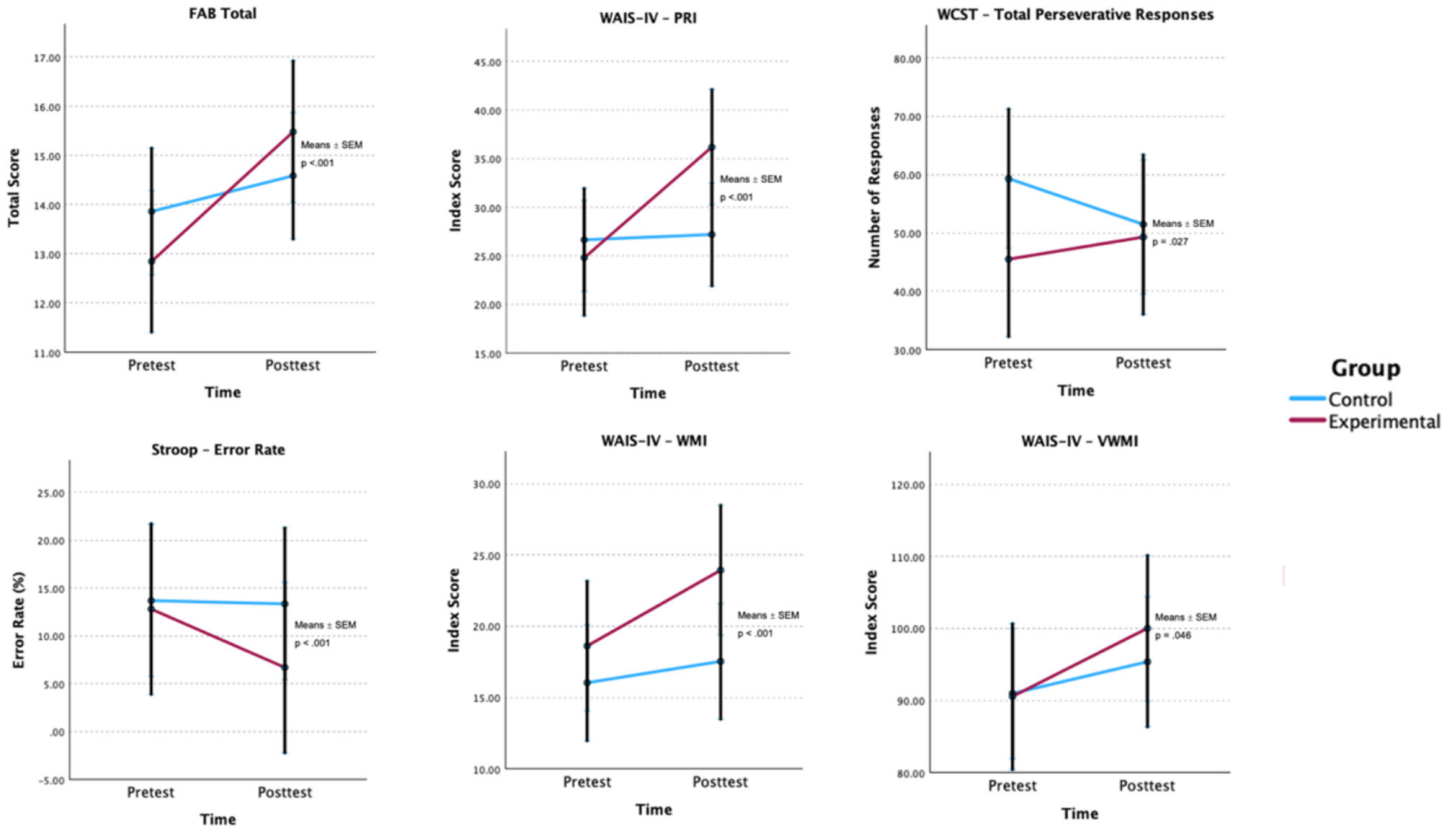
Estimated group × time effects for executive functioning outcomes. Estimated marginal means (EMMs) ± SEM are shown for each group at pre- and post-test. *p*-values are Bonferroni-adjusted. FAB, Frontal Assessment Battery; PRI, Perceptual Reasoning Index; WCST, Wisconsin Card Sorting Task; WAIS-IV, Wechsler Adult Intelligence Scale—Fourth Edition; WMS-IV, Wechsler Memory Scale—Fourth Edition; VWMI, Visual Working Memory Index.

The results of adjusted GLMM models were used with relevant covariates, such as age, clinical diagnosis, duration of drug use, marital status, and type of substances (Table 2). A significant interaction between group and time was observed for FAB Total [*F*_(1, 75)_ = 20.05, *p* < 0.001], PRI Index (WAIS-IV) [*F*_(1, 75)_ = 49.26, *p* < 0.001], Total Perseverative Responses (WCST) [*F*_(1, 75)_ = 5.06, *p* = 0.027], Error Rate (Stroop) [*F*_(1, 75)_ = 25.39, *p* < 0.001], WMI Index (WAIS-IV) [*F*_(1, 75)_ = 16.11, *p* < 0.001], and VWMI Index (WMS-IV) [*F*_(1, 75)_ = 4.14, *p* = 0.046] (Figure 2), indicating improvements in overall executive functioning, perceptual reasoning, cognitive flexibility, inhibitory control, and working memory in the experimental group compared to the control group. It is also important to note that a significant group × time interaction was also found for Total Perseverative Responses (WCST) [*F*_(1, 75)_ = 5.06, *p* = 0.027]; however, these improvements in cognitive flexibility were observed in the control group, not the experimental group.

**Table 2:** Estimated marginal means and GLMM analysis of executive functioning measures: group × time interaction and covariate-adjusted effects.

	**CG**		**EG**		**Interaction Time** × **group**		**Effect of age**		**Effect of clinical diagnosis**		**Effect of drug use time**		**Effect of marital status**		**Effect of substance type**	
	**Pre-test**	**Post-test**	**Pre-test**	**Post-test**												
**Variables**	***M*** **[95% CI]**	***M*** **[95% CI]**	***M*** **[95% CI]**	***M*** **[95% CI]**	***F*** **(df)**	***p***	***F*** **(df)**	***p***	***F*** **(df)**	***p***	***F*** **(df)**	***p***	***F*** **(df)**	***p***	***F*** **(df)**	***p***
**Executive functioning**																
**Generalized executive functioning**																
FAB total	13.86 [12.57–15.15]	14.59 [13.30–15.88]	12.84 [11.40–14.29]	15.48 [14.04–16.93]	20.05 (1, 75)	**<0.001**	3.98 (1, 75)	**0.050**	3.76 (1, 75)	**0.004**	5.06 (1, 75)	**0.027**	5.99 (1, 75)	**0.004**	1.52 (1, 75)	0.184
**Perceptual reasoning**																
PRI index (WAIS-IV)	26.64 [21.34–31.94]	27.19 [21.88–32.49]	24.79 [18.86–30.72]	36.19 [30.26–42.12]	49.26 (1, 75)	**<0.001**	0.46 (1, 75)	0.501	0.84 (1, 75)	0.525	0.50 (1, 75)	0.482	1.05 (1, 75)	0.354	0.62 (1, 75)	0.715
**Cognitive flexibility**																
Time (TMT B)	60.61 [34.53–86.69]	57.75 [31.67–83.83]	61.73 [32.52–90.95]	49.85 [20.64–79.07]	1.92 (1, 75)	0.171	0.50 (1, 75)	0.481	0.33 (1, 75)	0.896	1.16 (1, 75)	0.286	0.47 (1, 75)	0.628	0.56 (1, 75)	0.762
Total perseverative responses (WCST)	59.33 [47.37–71.30]	51.47 [39.51–63.43]	45.49 [32.23–58.75]	49.29 [36.03–62.55]	5.06 (1, 75)	**0.027**	0.15 (1, 75)	0.702	1.04 (1, 75)	0.403	0.01 (1, 75)	0.942	1.35 (1, 75)	0.265	1.65 (1, 75)	0.145
**Inhibitory control**																
Error rate (Stroop)	13.70 [5.75–21.66]	13.36 [5.41–21.31]	12.81 [3.88–21.75]	6.70 [−2.23–15.64]	25.39 (1, 75)	**<0.001**	0.29 (1, 75)	0.589	0.61 (1, 75)	0.694	1.17 (1, 75)	0.282	0.19 (1, 75)	0.826	0.63 (1, 75)	0.710
Interference effect (Stroop)	74.38 [−11.46–160.22]	77.91 [−7.93–163.75]	54.88 [−39.45–149.22]	58.94 [−35.39–153.28]	0.00 (1, 75)	0.991	1.76 (1, 75)	0.188	0.89 (1, 75)	0.495	0.17 (1, 75)	0.680	0.78 (1, 75)	0.464	1.23 (1, 75)	0.302
**Working memory**																
WMI index (WAIS-IV)	16.03 [11.97–20.10]	17.53 [13.47–21.60]	18.61 [14.06–23.17]	23.93 [19.38–28.49]	16.11 (1, 75)	**<0.001**	0.95 (1, 75)	0.332	0.33 (1, 75)	0.895	0.10 (1, 75)	0.752	0.35 (1, 75)	0.705	0.37 (1, 75)	0.894
**Visual working memory**																
VWMI index (WMS-IV)	90.96 [81.91–100.02]	95.37 [86.32–104.43]	90.55 [80.41–100.68]	100.03 [89.89–110.16]	4.14 (1, 75)	**0.046**	0.31 (1, 75)	0.581	1.20 (1, 75)	0.963	0.52 (1, 75)	0.473	0.00 (1, 75)	0.997	0.93 (1, 75)	0.478

FAB, Frontal Assessment Battery; PRI Index, Perceptual Reasoning Index; WAIS-IV, Wechsler Adult Intelligence Scale—Fourth Edition; TMT B, Trail Making Test Part B; WCST, Wisconsin Card Sorting Task; WMS-IV, Wechsler Memory Scale—Fourth Edition; VWMI Index, Visual Working Memory Index; p, Significance Level; EG, Experimental Group; CG, Control Group.

Bold values indicate statistically significant results (*p* < 0.05).

In addition to the significant effect of the time × group interaction on Total FAB, the GLMM model revealed statistically relevant effects of several covariates on the variable in question. A positive association was found between total FAB performance and age, indicating that older participants performed slightly better on generalized executive functioning (*t* = 2.00, *p* = 0.050). Significant differences were also observed in clinical diagnosis, with participants having cocaine dependence (*t* = 2.19, *p* = 0.032) and opioid dependence (*t* = 2.61, *p* = 0.011) scoring higher on the FAB Total. Conversely, the duration of substance use demonstrated a significant negative effect (*t* = −2.25, *p* = 0.027), suggesting that longer duration of use is associated with poorer cognitive performance in this domain. Regarding marital status, a statistically significant negative effect was identified for divorced participants (*t* = −2.98, *p* = 0.004), indicating poorer performance in generalized executive functioning among these participants.

Notable differences were observed between the pre- and post-test of the groups on certain cognitive measures, with the CG scoring higher than the EG in the FAB Total, PRI Index, Total Perseverative Responses of the WCST, and Stroop Interference Effect in the pre-test. These differences were addressed in the statistical analysis by including covariates in the adjusted GLMM models. Other measures did not show significant differences, specifically TMT-B Time, Stroop Error Rate, and VWMI Index.

However, in the post-test, the EG showed improvements in several measures, namely in the FAB Total, which increased by 2.64 points (95% CI [0.49, 4.79]) in the EG compared to the CG, which only increased by 0.73 points (95% CI [−1.21, 2.67]). The same was true for other measures, namely the PRI Index (GE: +11.40, 95% CI [1.09, 5.34]), WMI Index (GE: +5.32, 95% CI [−1.46, 12.10]), and VWMI Index (GE: +9.48, 95% CI [−5.61, 24.57]). There was also a greater reduction in the Stroop Error Rate in the EG (−6.11; 95% CI [−17.87, 5.65]) and the total perseverative responses of the WCST (+3.80; 95% CI [−11.66, 4.06]).

#### 3.2.2 Memory

**Figure 3:**
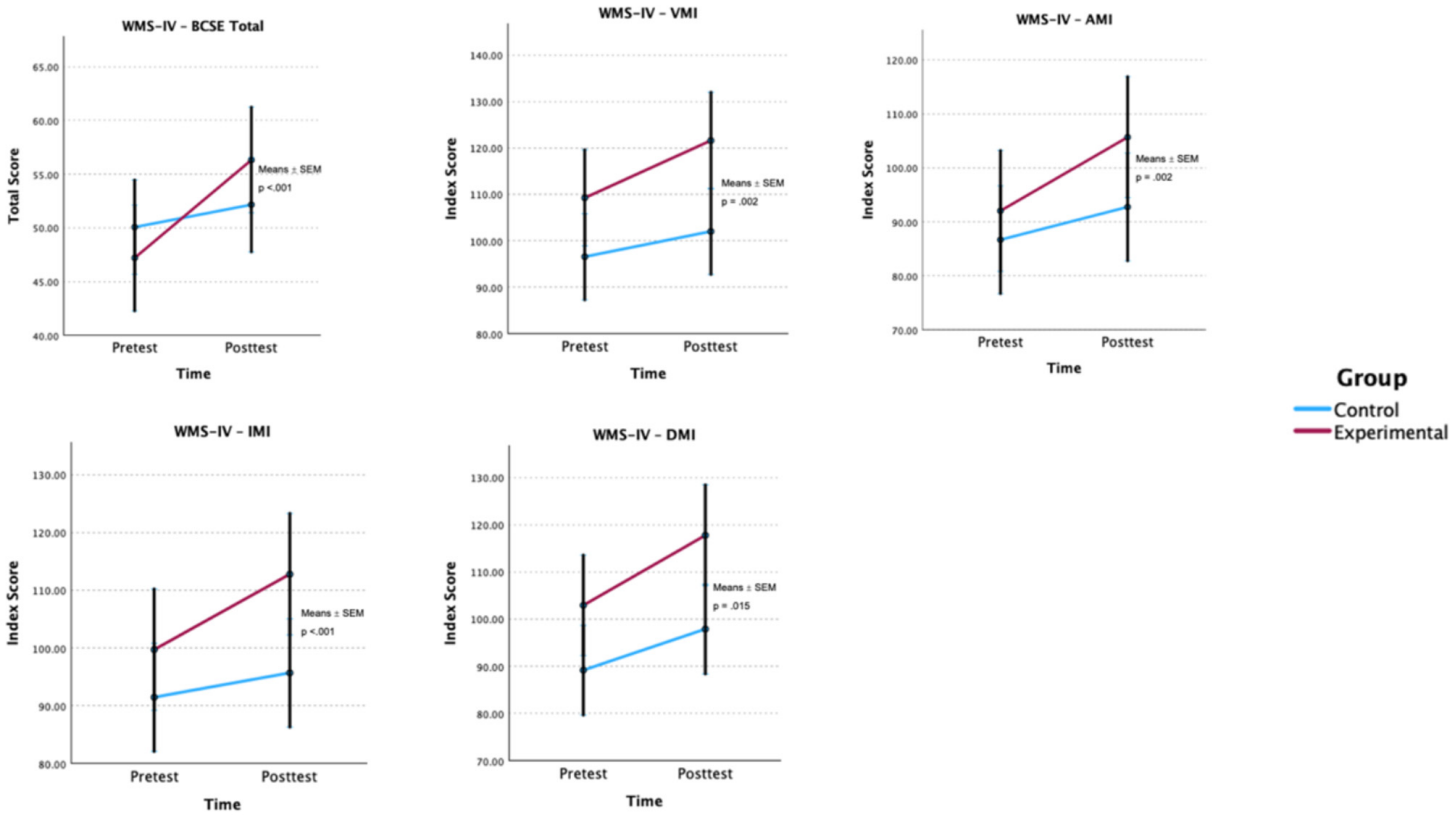
Estimated group × time effects for memory outcomes. Estimated marginal means (EMMs) ± SEM are shown for each group at pre- and post-test. *p*-values are Bonferroni-adjusted. BCSE, Brief Cognitive Status Exam; VMI, Visual Memory Index; AMI, Auditory Memory Index; IMI, Immediate Memory Index; DMI, Delayed Memory Index; WMS-IV, Wechsler Memory Scale—Fourth Edition.

Similar to the approach used for executive functioning, and to thoroughly assess the intervention's effectiveness, the results from the adjusted GLMM models were also applied to memory measures (see Table 3). A significant group × time interaction with covariates such as age, clinical diagnosis, duration of drug use, marital status, and substance type was observed for all WSM memory variables, including BCSE Total [*F*_(1, 75)_ = 36.42, *p* < 0.001], VMI Index [*F*_(1, 75)_ = 10.52, *p* = 0.002], AMI Index [*F*_(1, 75)_ = 10.84, *p* = 0.002], IMI Index [*F*_(1, 75)_ = 20.68, *p* < 0.001], and DMI Index [*F*_(1, 75)_ = 6.26, *p* = 0.015] (Figure 3), suggesting that the magnitude of change differed between groups, with greater improvements in general memory functioning, visual memory, auditory memory, and both immediate and delayed memory in the experimental group.

**Table 3:** Estimated marginal means and GLMM analysis of memory measures: group × time interaction and covariate-adjusted effects.

	**CG**		**EG**		**Interaction Time** × **group**		**Effect of age**		**Effect of clinical diagnosis**		**Effect of drug use time**		**Effect of marital status**		**Effect of substance type**	
	**Pre-test**	**Post-test**	**Pre-test**	**Post-test**												
**Variables**	***M*** **[95% CI]**	***M*** **[95% CI]**	***M*** **[95% CI]**	***M*** **[95% CI]**	***F*** **(df)**	***p***	***F*** **(df)**	***p***	***F*** **(df)**	***p***	***F*** **(df)**	***p***	***F*** **(df)**	***p***	***F*** **(df)**	***p***
**Memory**																
**Global memory**																
BCSE total (WMS-IV)	50.06 [45.66–54.46]	52.15 [47.75–56.55]	47.19 [42.27–52.12]	56.31 [51.39–61.24]	36.42 (1, 75)	**<0.001**	0.51 (1, 75)	0.479	0.13 (1, 75)	0.350	1.84 (1, 75)	0.179	1.39 (1, 75)	0.255	1.41 (1, 75)	0.221
**Visual memory**																
VMI index (WMS-IV)	96.54 [87.28–105.80]	102.00 [92.73–111.26]	109.23 [98.85–119.61]	121.59 [111.21–131.97]	10.52 (1, 75)	**0.002**	2.70 (1, 75)	0.105	1.03 (1, 75)	0.405	0.91 (1, 75)	0.344	2.41 (1, 75)	0.096	0.24 (1, 75)	0.961
**Auditory memory**																
AMI Index (WMS-IV)	86.66 [76.66–96.66]	92.75 [82.75–102.75]	92.03 [80.81–103.24]	105.71 [94.49–116.92]	10.84 (1, 75)	**0.002**	0.32 (1, 75)	0.572	1.16 (1, 75)	0.338	0.13 (1, 75)	0.719	3.42 (1, 75)	**0.038**	1.02 (1, 75)	0.419
**Immediate memory**																
IMI Index (WMS-IV)	91.45 [82.07–100.83]	95.68 [86.29–105.06]	99.71 [89.18–110.23]	112.79 [102.26–123.31]	20.68 (1, 75)	**<0.001**	1.40 (1, 75)	0.241	1.00 (1, 75)	0.422	0.28 (1, 75)	0.596	3.13 (1, 75)	**0.050**	0.97	0.455
**Delayed memory**																
DMI Index (WMS)	89.15 [79.63–98.68]	97.88 [88.35–107.402]	102.92 [92.25–113.58]	117.76 [107.09–128.42]	6.26 (1, 75)	**0.015**	1.99 (1, 75)	0.162	0.70 (1, 75)	0.629	1.35 (1, 75)	0.249	2.36 (1, 75)	0.101	0.42 (1, 75)	0.861

BCSE, Brief Cognitive Status Exam; WMS-IV, Wechsler Memory Scale—Fourth Edition; VMI Index, Visual Memory Index; AMI Index, Auditory Memory Index; IMI Index, Immediate Memory; DMI Index, Delayed Memory Index; p, Significance Level; EG, Experimental Group; CG, Control Group.

Bold values indicate statistically significant results (*p* < 0.05).

It should also be noted that in the pre-test, differences were observed between the groups in the memory variables. The EG demonstrated higher VMI Index, AMI Index, IMI Index, and DMI Index values, while the CP only scored higher in the pre-test for the BCSE Total.

In the post-test, the EG demonstrated greater improvements across all memory domains. The BCSE Total rose by 9.12 points (95% CI [1.79, 16.45]), the VMI Index increased by 12.36 points (95% CI [−3.10, 27.82]), the AMI Index improved by 13.68 points (95% CI [−3.02, 30.38]), the IMI Index went up by 13.08 points (95% CI [−2.59, 28.75]), and the DMI Index increased by 14.84 points (95% CI [−1.04, 30.72]).

A statistically significant negative correlation of weak magnitude was found between age and VMI Index (*r* = −0.03, *p* = 0.050), suggesting that increased age is associated with lower VMI scores. No significant correlations were observed for the other memory variables.

#### 3.2.3 Processing speed

**Figure 4:**
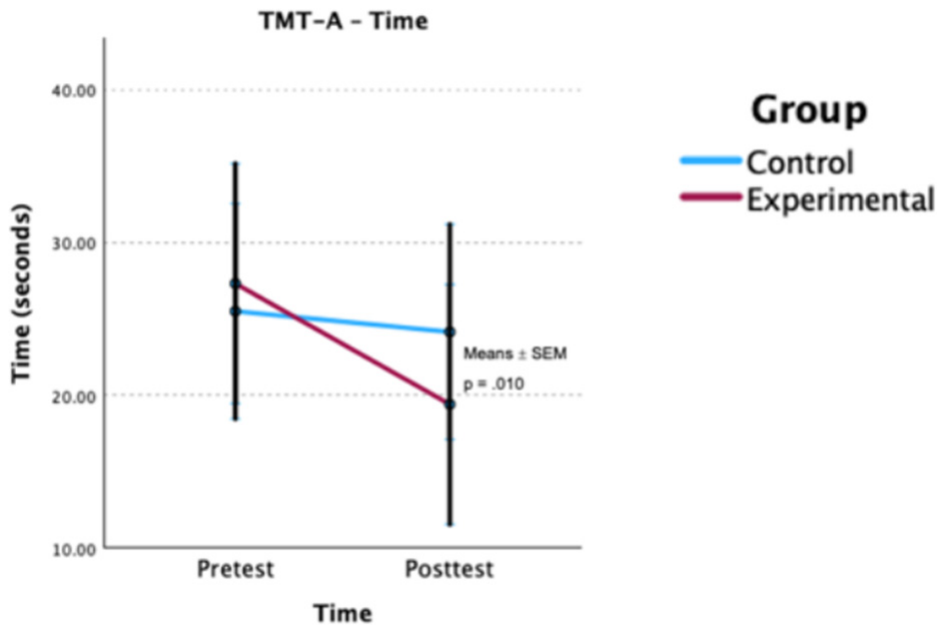
Estimated group × time effects for processing speed (TMT-A). Estimated marginal means (EMMs) ± SEM are shown for each group at pre- and post-test. *p*-value is Bonferroni-adjusted. TMT-A, Trail Making Test—Part A.

Using GLMM with the same covariates identified previously (Table 4), a significant group × time interaction was observed for TMT-A completion time [*F*_(1, 75)_ = 6.93, *p* = 0.010] (Figure 4). The experimental group demonstrated greater improvement in average completion time following the intervention, with a mean reduction of 7.92 s, compared to a 1.14-s reduction observed in the control group. However, when considering age, clinical diagnosis, duration of drug use, marital status, and substance type as covariates, no statistically significant effects were observed for TMT-A completion time (*p* > 0.05). Additionally, no significant interaction effects were noted for PSI Index (WAIS-IV; *p* > 0.05).

**Table 4:** Estimated marginal means and GLMM analysis of processing speed measures: group × time interaction and covariate-adjusted effects.

	**CG**		**EG**		**Interaction Time** × **group**		**Effect of age**		**Effect of clinical diagnosis**		**Effect of drug use time**		**Effect of marital status**		**Effect of substance type**	
	**Pre-test**	**Post-test**	**Pre-test**	**Post-test**												
**Variables**	***M*** **[95% CI]**	***M*** **[95% CI]**	***M*** **[95% CI]**	***M*** **[95% CI]**	***F*** **(df)**	***p***	***F*** **(df)**	***p***	***F*** **(df)**	***p***	***F*** **(df)**	***p***	***F*** **(df)**	***p***	***F*** **(df)**	***p***
**Processing speed**																
Time (TMT A)	25.49 [18.43–32.55]	24.13 [17.07–31.19]	27.31 [19.44–35.18]	19.39 [11.52–27.26]	6.93 (1, 75)	**0.010**	0.21 (1, 75)	0.648	0.24 (1, 75)	0.944	0.59 (1, 75)	0.445	0.29 (1, 75)	0.752	0.43 (1, 75)	0.860
PSI index (WAIS-IV)	18.71 [14.23–23.20]	20.95 [16.45–25.44]	21.30 [16.29–26.31]	25.74 [20.73–30.75]	2.45 (1, 74)	0.122	1.00 (1, 74)	0.320	0.702 (1, 74)	0.624	0.09 (1, 74)	0.766	0.54 (1, 74)	0.586	0.63 (1, 75)	0.702

TMT A, Trail Making Test Part A; WAIS-IV, Wechsler Adult Intelligence Scale, Fourth Edition; PSI Index, Processing Speed Index; p, Significance Level; EG, Experimental Group; CG, Control Group.

Bold values indicate statistically significant results (*p* < 0.05).

It is also important to note that some differences were identified between the groups in terms of processing speed variables in the pre-test. The EG had a longer average time on the TMT-A, indicating poorer performance for this group, and slightly higher values on the WAIS-IV PSI Index. However, these differences were considered by including covariates in the adjusted GLMM models.

In the post-test, there was an improvement in processing speed for the EG, with an average reduction of 7.92 s in the TMT-A (95% CI [−19.64, 3.80]) and an increase of 4.44 points in the PSI Index (95% CI [−3.02, 11.90]).

A statistically significant negative correlation of low to moderate magnitude was found between age and PSI Index (ρ = −0.36, *p* = 0.013), suggesting that increased age is associated with lower PSI scores. Furthermore, a statistically significant negative correlation of moderate magnitude was found between time of use and PSI Index (ρ = −0.30, *p* = 0.041), suggesting that a longer history of substance use is associated with lower PSI scores. No other significant correlations were observed for processing speed.

### 3.3 Secondary outcomes

#### 3.3.1 False memories

Using GLMM with the same previous covariates (see Table 5), a significant group × time interaction was observed for False Recall Critical Lures (DRM) [*F*_(1, 74)_ = 4.03, *p* = 0.048]. The experimental group demonstrated a notable reduction in false recall of critical lures following the intervention, with an average decrease of 1.8 words. In contrast, the control group showed a slight increase of 0.63 words.

**Table 5:** Estimated marginal means and GLMM analysis of false memory measures: group × time interaction and covariate-adjusted effects.

	**CG**		**EG**		**Interaction Time** × **group**		**Effect of age**		**Effect of clinical diagnosis**		**Effect of drug use time**		**Effect of marital status**		**Effect of substance type**	
	**Pre-test**	**Post-test**	**Pre-test**	**Post-test**												
**Variables**	***M*** **[95% CI]**	***M*** **[95% CI]**	***M*** **[95% CI]**	***M*** **[95% CI]**	***F*** **(df)**	***p***	***F*** **(df)**	***p***	***F*** **(df)**	***p***	***F*** **(df)**	***p***	***F*** **(df)**	***p***	***F*** **(df)**	***p***
**False memories**																
False recall critical lures (DRM)	6.27 [3.09–9.45]	6.92 [3.73–10.11]	8.53 [5.00–12.06]	6.73 [3.20–10.26]	4.03 (1, 74)	**0.048**	0.03 (1, 74)	0.865	0.25 (1, 74)	0.937	0.70 (1, 74)	0.404	0.98 (1, 74)	0.381	0.41 (1, 74)	0.870
False recognition critical lures (DRM)	12.29 [8.82–15.77]	13.86 [10.39–17.34]	13.30 [9.42–17.17]	13.34 [9.46–17.21]	2.46 (1, 74)	0.121	1.07 (1, 74)	0.304	0.55 (1, 74)	0.741	2.83 (1, 74)	0.096	0.53 (1, 74)	0.589	2.02 (1, 74)	0.074
Total confabulation (CB)	3.67 [2.22–5.12]	2.53 [1.08–3.98]	2.80 [1.18–4.42]	1.68 [0.06–3.30]	0.00 (1, 75)	0.975	0.10 (1, 75)	0.758	5.05 (1, 75)	**<0.001**	4.21 (1, 75)	**0.044**	1.86 [1, 75]	0.163	2.77 (1, 75)	**0.017**

DRM, Deese-Roediger-McDermott Paradigm; CB, Dalla Barba Confabulation Battery; p, Significance Level; EG, Experimental Group; CG, Control Group.

Bold values indicate statistically significant results (*p* < 0.05).

In the DRM False Recognition of Unrelated Lures, although there were no statistically significant differences in the interaction between group and time [*F*_(1, 74)_ = 0.33, *p* = 0.568], there was a significant effect on the variables age [*F*_(1, 74)_ = 9.23, *p* = 0.003] and time of drug use [*F*_(1, 74)_ = 7.18, *p* = 0.009]. The analysis of the fixed coefficients showed a significant positive effect (*t* = 3.04, *p* = 0.003), indicating that as participants age, their scores in False Recognition of Unrelated Lures tend to increase. However, the time of drug use showed a negative effect (*t* = −2.68, *p* = 0.009), suggesting that the longer the consumption time, the lower the score on this subtest of the DRM.

Regarding total confabulations, although there was no statistically significant effect in the interaction between time and group [*F*_(1, 75)_ = 0.00, *p* = 0.975], the model with the covariates showed a significant effect for clinical diagnosis [*F*_(1, 75)_ = 5.05, *p* < 0.001], drug use time [*F*_(1, 75)_ = 4.21, *p* = 0.044] and substance type [*F*_(1, 75)_ = 2.77, *p* = 0.017]. The analysis of fixed coefficients indicated a positive effect (*t* = 2.143, *p* = 0.035), suggesting that individuals with a longer duration of drug use tend to have a greater number of confabulations, regardless of the type of intervention, age, or clinical diagnosis.

The analysis of various types of confabulations using fixed coefficients revealed a positive effect on misplacement-type confabulation (*t* = 2.68, *p* = 0.009) and memory confusion (*t* = 3.15, *p* = 0.002). This suggests that individuals who have used drugs for longer exhibit a greater number of confabulations of this type.

It should be noted that in the pre-test, the EG exhibited slightly higher values than the CG in the DRM paradigm tasks and overall confabulation. These differences were considered in the GLMM models and adjusted for false memories.

In the post-test, the EG experienced a decrease of 1.80 points in False Recall Critical Lures (95% CI [−7.06, 3.46]), whereas the CG showed a small increase. For False Recognition Critical Lures and total confabulations, both groups had identical results with no significant differences between the pre- and post-test.

Conversely, the linear regression analysis indicated that enhancements in executive functioning did not differentially impact false memories between groups. In the EG, individuals who showed improvements in general executive functioning (Total FAB) did not show a reduction in episodic confabulations as measured by CB (regression coefficient (B) = 0.03, *p* = 0.382, 95% bias-corrected and accelerated confidence interval (CI BCa) [−0.22;0.39]), nor in spontaneous false memories through the DRM paradigm (B = −0.85, *p* = 0.365, 95% CI BCa [−0.28;0.42]), compared to the CG. On the other hand, participants in the EG who experienced greater improvements in memory, especially in delayed memory, showed a tendency toward fewer episodic confabulations (B = −0.05, *p* = 0.037, 95% CI [−0.11; −0.01]). However, this trend did not hold for spontaneous false memories (B = 0.20, *p* = 0.193, 95% CI [−0.06;0.53]).

## 4 Discussion

Cognitive impairments can hinder the effectiveness of psychosocial treatments, which are primary interventions for SUD. Enhancing cognitive function can improve responses to these treatments and lead to better treatment outcomes (Nardo et al., 2022; Sofuoglu et al., 2013). The present study evaluated the effectiveness of a VR-based cognitive training platform, VRainSUD-VR, as a complement to standard residential treatment for patients with SUD. The results generally supported that adding the VRainSUD-VR program to the standard treatment improved overall cognitive functioning, specifically in executive functions and memory. Furthermore, it showed promising results in reducing the formation of false memories.

Regarding treatment outcomes, the experimental group had an 8% dropout rate compared to 27% in the control group. While this difference was not statistically significant, it may represent a clinically meaningful benefit by encouraging greater treatment retention. Moreover, improvements in executive function and memory may hold clinical relevance, as deficits in these domains are associated with poorer treatment adherence, greater emotion regulation difficulties, and increased relapse vulnerability in SUD (Rolland et al., 2019; Verdejo-Garcia et al., 2019). Therefore, improvements in executive functioning and memory may contribute to improved treatment engagement and long-term recovery outcomes.

The cognitive training intervention (see GLMM statistical analysis) had a positive impact on participants' general executive functioning, specifically in perceptual reasoning, working memory, and visual working memory, albeit with some reservations regarding cognitive flexibility and inhibitory control.

Regarding general executive functioning, the FAB is a standard measure for understanding overall impairment in this cognitive domain among individuals with SUD (Cunha et al., 2010). The results indicate that adding VRainSUD-VR to TAU led to greater improvements compared to TAU alone. In addition to the main effect of time × group on FAB Total, the analysis revealed that certain covariates influenced executive functioning. On one hand, older participants and those with a diagnosis of cocaine or opioid dependence appeared to benefit more from the intervention. Cognitive training has been shown to improve executive functioning in older adults without known cognitive impairment (Nguyen et al., 2019), and the primary drug of use may have differential effects on this cognitive domain (Van Der Plas et al., 2009). Conversely, poorer overall performance in executive functioning was associated with a longer history of substance use. Chronic and heavy use of substances is consistently linked to significant impairments in executive functions (Hagen et al., 2016), and further research is needed to understand the role of marital status in cognitive performance. These findings suggest that responsiveness to cognitive interventions aimed at executive functioning may depend on individual characteristics and their interaction.

Adding VRainSUD-VR to TAU was associated with significant improvements in perceptual reasoning. Although studies indicate that cognitive training can enhance reasoning in various populations, including children (Joseph et al., 2017; Mackey et al., 2011), adults (Boron et al., 2007; Schmiedek et al., 2014), and the elderly (Corbett et al., 2015; Willis and Schaie, 1986), the research specifically focusing on perceptual reasoning is limited. A study involving young adults who underwent 101 daily cognitive training sessions observed significant improvements in reasoning, and these gains were maintained even after 2 years (Schmiedek et al., 2014). More studies investigating perceptual reasoning are needed, including long-term follow-up studies, both in the context of substance abuse and in other populations.

Working memory demonstrated significant improvements, including in the visual domain, with results supporting the effectiveness of VRainSUD-VR. These findings are consistent with the study by Towe et al. (2021). They concluded that participants in the experimental group who underwent web-based cognitive training focused on working memory showed significant reductions in working memory deficits compared to the control group, particularly for individuals with human immunodeficiency virus (HIV) and cocaine use disorder. Furthermore, Rass et al. (2015) found that working memory training in methadone maintenance patients led to improved performance on working memory tasks and reduced frequency of drug use. Similar results were found by Khemiri et al. (2019) in individuals with alcohol use disorder, where, after 5 weeks of working memory training, the experimental group showed significant improvements, primarily in verbal working memory.

Cognitive flexibility generally improves after treatment, although only the Total Perseverative Responses on the WCST showed significant changes in the experimental group. A likely reason for this finding is that the TMT and WSCT may not be sensitive enough to detect neurocognitive alterations in this population. A study with a large sample of patients receiving SUD treatment (*N* = 8,521) revealed that most performed within normal limits on the TMT (Roberts and Horton, 2001). Furthermore, a study by Cunha et al. (2010) reveals that there are no significant differences in baseline measures of Stroop and WSCT for individuals with SUD compared to healthy controls. Therefore, the potential to benefit from the intervention may be limited, as participants with higher initial abilities may experience less improvement compared to those with lower baseline performance (Traut et al., 2021). Gamito et al. (2021) support our mixed results regarding the partial effectiveness of cognitive training for cognitive flexibility. In their study of cognitive training with VR exercises based on activities of daily living, they found a significant group × time interaction in one out of six WCST outcomes (the number of errors) for the experimental group.

Inhibitory control yielded mixed results. Marceau et al. (2017) demonstrated that women with substance abuse who underwent cognitive remediation showed improvements in inhibitory control, as assessed by the Color-Word Interference Test. In another study (Pennington et al., 2022), a sample of veterans with alcohol use disorder and a history of traumatic brain injury found that the experimental group receiving executive function training through virtual reality, combined with physical exercise, achieved improvements in inhibitory control. Furthermore, Brooks et al. (2017) demonstrated that working memory training improved self-regulation and reduced impulsivity in methamphetamine users, highlighting its potential to enhance treatment outcomes. However, in the context of substance abuse, few studies have demonstrated improvements specifically in this domain, with most studies referring to executive functioning in general (Anderson et al., 2023; Gamito et al., 2014, 2017; Verdejo-Garcia et al., 2019), which encompasses inhibitory control. Inhibitory control involves distinct cognitive processes, and the tasks used to assess it may differ in their sensitivity to both the intervention and their specific cognitive demands (Hung et al., 2018; Tiego et al., 2018). Future studies should investigate how different tasks affect specific components of inhibitory control, namely response inhibition and attentional inhibition.

Memory impairments are common in SUD across various substances, including alcohol, cocaine, and methamphetamines, affecting both verbal and visual memory (Li et al., 2020; Potvin et al., 2018; Verdejo-Garcia et al., 2019). The memory domain exhibited the most positive results, with all measured variables indicating the effectiveness of the VRainSUD-VR program. The current findings align with previous research. These results support Steingass et al. (1994), who found that cognitive training leads to improvements in memory, particularly verbal memory, among individuals with alcohol dependence. A more recent study (Bell et al., 2016) of war veterans with alcohol use disorder demonstrated that the group receiving cognitive training daily for 13 weeks showed significant improvements in verbal memory.

Mixed results were observed for processing speed: while a significant effect was found for TMT-A, no significant changes were observed for PSI. Our results corroborate those reported in the systematic review by Caetano et al. (2021), which concluded that, among all the studies included, only one showed significant improvements in this area (Rezapour et al., 2019). The findings suggest that improvements in processing speed may require a longer or more intensive intervention, or that potential effects might only become apparent at follow-up. Höijer et al. (2020) conducted a neuropsychological evaluation in patients with SUD and mood disorders, finding that individuals with this comorbidity had greater deficits in processing speed compared to those with SUD alone. To minimize confounding variables that could interfere with cognitive performance, individuals with major depressive disorders were excluded from our sample. Given this, it is possible that processing speed may not represent a primary target for intervention in individuals with SUD without other psychiatric comorbidities.

Consistent with prior findings, older age was associated with decreased performance on tasks measuring visual memory and processing speed (Eckert, 2011; Tröndle and Langer, 2024). Additionally, a longer duration of drug use was associated with decreased performance on tasks measuring processing speed. These findings align with the growing evidence suggesting that chronic substance use may have cumulative neurocognitive effects (Hanson et al., 2011; Liu et al., 2022), underscoring the potential long-term cognitive consequences of continuous drug use.

Substance use contributes to the increasing susceptibility to false memories. We found a significant reduction in the false recall of critical lures in the experimental group post-intervention. Critical lures are typically recognized with high confidence (Pardilla-Delgado and Payne, 2017), and their reduction suggests that the VRainSUD-VR program effectively minimized susceptibility to false memories. Although modest in absolute terms (1.8 words), this improvement may still reflect meaningful gains in source monitoring, which is clinically relevant for individuals with SUD. Additionally, we found a significant association between improvements in delayed memory and a decrease in episodic confabulations. This finding indicates that improvements in delayed memory may assist in retrieving past experiences more accurately, thereby reducing the tendency to fill memory gaps with fabricated or inaccurate information. Previous studies underscore that confabulations are related to deficits in episodic memory retrieval (e.g., Dalla Barba, 1993), which highlights the potential of memory-based interventions to mitigate confabulatory behavior. The VRainSUD-VR program may strengthen hippocampal–prefrontal connectivity, enhancing episodic binding and top-down monitoring processes that reduce the likelihood of semantically driven false memories (Eichenbaum, 2017). Nonetheless, the mechanisms by which VR-based cognitive training influences false memory remain unclear. Repetitive tasks may help reduce false memories (Shao et al., 2022), and VR could enhance source monitoring by offering immersive, context-rich environments that support accurate memory attribution. However, VR might also blur the line between real and virtual experiences, potentially increasing source confusion (Rubo et al., 2021). Future studies should examine false memory as a primary outcome and compare VR with other training formats to clarify these effects.

Older participants were more susceptible to false memories associated with items that were neither previously presented nor semantically related. While the production of false memories can be observed across all age groups, their frequency and contextual richness tend to increase with age (Abichou et al., 2020). The duration of drug use showed mixed results regarding its impact on false memories. Contrary to expectations, DRM results indicated that a longer duration of drug use was associated with reduced susceptibility to false memories tied to items that were neither presented before nor semantically related. However, a longer duration of drug use was linked to confabulations in the CB. This finding may be due to unmeasured variables not included in our model, such as frequency or patterns of drug use, which could significantly impact these outcomes. Furthermore, distinct cognitive mechanisms may underlie susceptibility to different types of memory errors. For example, false memories may involve source or semantic processing errors, whereas confabulations might reflect monitoring or executive deficits. Thus, longer drug use might specifically affect reality monitoring without increasing susceptibility to all types of false memories.

This study has several limitations that should be acknowledged. First, it focused on cognitive outcomes that are indirectly related to treatment success but did not include post-intervention clinical measures that could provide more direct indicators of the intervention's effectiveness. Second, without a follow-up assessment, it remains unknown whether the cognitive improvement effect has been sustained over long periods. Third, the use of time-consuming and extensive neuropsychological batteries and tests may limit feasibility and increase participant burden, especially in pre- and post-intervention assessments. Fourth, the lack of an active control group (e.g., paper-based or neutral VR training) limits attribution of effects to the VR intervention itself, as engagement or novelty may have played a role. Previous studies have included active control groups for comparisons with the experimental group (Man, 2020). However, implementing an active control within the treatment units was not feasible due to logistical constraints, including small clinical teams, the need to maintain uniform care, and the additional training that would have been required. This methodological choice is acknowledged as a limitation in terms of internal validity and generalizability. Fifth, the higher dropout rate in the control group, though not statistically significant, may indicate differential motivation, but the reasons were not systematically recorded. Another important limitation is the presence of significant baseline differences between groups in memory measures. Although these were statistically controlled in the model, they may have influenced the results, limiting the interpretation of the intervention effects. Moreover, conducting the intervention within a single clinical unit might have introduced site-related bias that could not be controlled. While a non-randomized design introduces selection bias, randomization was not feasible because selectively delivering the VR intervention within the same unit risked participant dissatisfaction and unequal treatment exposure. Furthermore, the staff conducting outcome assessments were not blinded to group allocation, which may have introduced bias despite the use of standardized instruments. Finally, adherence to VR sessions and potential adverse effects, including cybersickness, were not monitored in this study, representing an additional limitation.

Future studies should include active control groups (e.g., non-VR cognitive training or neutral VR exposure) to identify the specific contribution of the VRainSUD-VR intervention and improve the methodological rigor of the study design. Furthermore, incorporating post-intervention clinical outcomes and follow-up assessments could allow for assessment of the relationship between cognitive improvements and relapse rates, as well as other clinical measures, thereby making it easier to recognize the intervention's clinical relevance. Moreover, future research should include compliance metrics and a systematic assessment of adverse events to improve the evaluation of feasibility and safety.

Since the statistical power of the sample was slightly <0.80, future studies with larger samples could help lower the risk of a type II error and strengthen the reliability of the findings.

## 5 Conclusion

Our results indicated that the VR-based cognitive training program VRainSUD-VR can effectively improve overall cognitive function, particularly memory and, to some extent, executive functioning, in individuals with SUD. Addressing neurocognitive functions may improve treatment benefits, suggesting that VRainSUD-VR could be integrated into residential programs to augment cognitive rehabilitation alongside standard care. Given the well-established role of cognitive impairments in reinforcing the addiction cycle and compromising treatment outcomes, further research is necessary to examine the long-term effects of this intervention. Specifically, randomized controlled trials (RCTs) with active control groups and follow-up assessments at 6 months (or longer) are essential to clarify its effectiveness. While this study targeted cognitive outcomes, future research should incorporate psychological mediators (e.g., emotion regulation) and clinical endpoints (e.g., relapse) to comprehensively evaluate treatment effectiveness.

## Data Availability

The raw data supporting the conclusions of this article will be made available by the authors, without undue reservation.

## References

[B1] Abadie M. Camos V. (2019). False memory at short and long term. J. Exp. Psychol. Gen. 148, 1312–1334. 10.1037/xge0000526 30550338

[B2] Abichou K. La Corte V. Nicolas S. Piolino P. (2020). False memory in normal ageing: empirical data from the DRM paradigm and theoretical perspectives. Gériatrie Psychologie Neuropsychiatrie Viellissement 18, 65–75. 10.1684/pnv.2020.0862 32576546

[B3] American Psychiatric Association (2013). Diagnostic and Statistical Manual of Mental Disorders, 5th Edn. Arlington, VA: American Psychiatric Publishing. 10.1176/appi.books.9780890425596

[B4] Anderson A. C. Robinson A. H. Giddens E. Hartshorn B. Allan E. Rowe C.. (2023). Proof-of-concept trial of Goal Management Training+ to improve executive functions and treatment outcomes in methamphetamine use disorder. Drug Alcohol Depend. 246:109846. 10.1016/j.drugalcdep.2023.109846 37004463

[B5] Babor T. F. Higgins-Biddle J. C. Saunders J. B. Monteiro M. G. (2001). The Alcohol Use Disorders Identification Test Guidelines for Use in Primary Care. Geneva: World Health Organization (WHO).vspace*0.5pt

[B6] Bauer A. C. M. Andringa G. (2020). The potential of immersive virtual reality for cognitive training in elderly. Gerontology 66, 614–623. 10.1159/000509830 32906122

[B7] Bell M. D. Vissicchio N. A. Weinstein A. J. (2016). Cognitive training and work therapy for the treatment of verbal learning and memory deficits in veterans with alcohol use disorders. J. Dual Diagn. 12, 83–89. 10.1080/15504263.2016.1145779 26828571 PMC4932894

[B8] Berman A. H. Bergman H. Palmstierna T. Schlyter F. (2003). The Drug Use Disorders Identification Test. Stockholm: Karolinska Institutet, Department of Clinical Neuroscience Section for Alcohol and Drug Dependence Research. 10.1037/t02890-000

[B9] Boron J. B. Turiano N. A. Willis S. L. Schaie K. W. (2007). Effects of cognitive training on change in accuracy in inductive reasoning ability. J. Gerontol. B 62, P179–P186. 10.1093/geronb/62.3.P179 17507586

[B10] Brainerd C. J. Reyna V. F. Ceci S. J. (2008). Developmental reversals in false memory: a review of data and theory. Psychol. Bull. 134, 343–382. 10.1037/0033-2909.134.3.343 18444700

[B11] Brandon T. H. Vidrine J. I. Litvin E. B. (2007). Relapse and relapse prevention. Annu. Rev. Clin. Psychol. 3, 257–284. 10.1146/annurev.clinpsy.3.022806.091455 17716056

[B12] Brooks S. J. Wiemerslage L. Burch K. H. Maiorana S. A. Cocolas E. Schiöth H.. (2017). The impact of cognitive training in substance use disorder: the effect of working memory training on impulse control in methamphetamine users. Psychopharmacology 234, 1911–1921. 10.1007/s00213-017-4597-6 28324119 PMC5486910

[B13] Brorson H. H. Ajo Arnevik E. Rand-Hendriksen K. Duckert F. (2013). Drop-out from addiction treatment: a systematic review of risk factors. Clin. Psychol. Rev. 33, 1010–1024. 10.1016/j.cpr.2013.07.007 24029221

[B14] Caetano T. Pinho M. S. Freire H. Mota D. Ramadas E. Lopes J.. (2025). VRainSUD: content validation of a cognitive training program using the Delphi method. Virtual Real. 29:105. 10.1007/s10055-025-01185-2

[B15] Caetano T. Pinho M. S. Ramadas E. Clara C. Areosa T. Dixe M. (2021). Cognitive training effectiveness on memory, executive functioning, and processing speed in individuals with substance use disorders: a systematic review. Front. Psychol. 12:730165. 10.3389/fpsyg.2021.730165 34489833 PMC8418081

[B16] Chisholm D. A. N. Doran C. Shibuya K. Rehm J. (2006). Comparative cost-effectiveness of policy instruments for reducing the global burden of alcohol, tobacco and illicit drug use. Drug Alcohol Rev. 25, 553–565. 10.1080/09595230600944487 17132573

[B17] Clemenson G. D. Stark C. E. L. (2015). Virtual environmental enrichment through video games improves hippocampal-associated memory. J. Neurosci. 35, 16116–16125. 10.1523/JNEUROSCI.2580-15.2015 26658864 PMC4682779

[B18] Cohen J. (1988). Statistical Power Analysis for the Behavioral Sciences, 2nd Edn. Hillsdale, NJ: Lawrence Erlbaum Associates.

[B19] Cohen J. (1992). A power primer. Psychol. Bull. 112, 155–159. 10.1037/0033-2909.112.1.155 19565683

[B20] Corbett A. Owen A. Hampshire A. Grahn J. Stenton R. Dajani S.. (2015). The effect of an online cognitive training package in healthy older adults: an online randomized controlled trial. J. Am. Med. Dir. Assoc. 16, 990–997. 10.1016/j.jamda.2015.06.014 26543007

[B21] Cunha P. J. Nicastri S. de Andrade A. G. Bolla K. I. (2010). The frontal assessment battery (FAB) reveals neurocognitive dysfunction in substance-dependent individuals in distinct executive domains: abstract reasoning, motor programing, and cognitive flexibility. Addict. Behav. 35, 875–881. 10.1016/j.addbeh.2010.05.005 20584570

[B22] Cunningham J. M. Pliskin N. H. Cassisi J. E. Tsang B. Rao S. M. (1997). Relationship between confabulation and measures of memory and executive function. J. Clin. Exp. Neuropsychol. 19, 867–877. 10.1080/01688639708403767 9524881

[B23] Dalla Barba G. (1993). Confabulation: knowledge and recollective experience. Cogn. Neuropsychol. 10, 1–20. 10.1080/02643299308253454

[B24] Dalla Barba G. Guerin B. Brazzarola M. Marangoni S. Barbera C. La Corte V. (2019). The confabulation battery: instructions and international data from normal participants. Neuropsychol. Rehabil. 29, 1625–1636. 10.1080/09602011.2018.1436446 29466921

[B25] Deese J. (1959). On the prediction of occurrence of particular verbal intrusions in immediate recall. J. Exp. Psychol. 58, 17–22. 10.1037/h0046671 13664879

[B26] Domínguez-Salas S. Díaz-Batanero C. Lozano-Rojas O. M. Verdejo-García A. (2016). Impact of general cognition and executive function deficits on addiction treatment outcomes: systematic review and discussion of neurocognitive pathways. Neurosci. Biobehav. Rev. 71, 772–801. 10.1016/j.neubiorev.2016.09.030 27793597

[B27] Drozdick L. W. Holdnack J. A. Hilsabeck R. C. (2011). Essentials of WMS-IV Assessment. Hoboken, NJ: John Wiley & Sons.

[B28] Dubois B. Slachevsky A. Litvan I. Pillon B. (2000). The FAB: a frontal assessment battery at bedside. Neurology 55, 1621–1626. 10.1212/WNL.55.11.1621 11113214

[B29] Eaton W. W. Smith C. Ybarra M. Muntaner C. Tien A. (2004). “Center for epidemiologic studies depression scale: review and revision (CESD and CESD-R),” in The Use of Psychological Testing for Treatment Planning and Outcomes Assessment: Instruments for Adults, 3rd Edn., Vol. 3, ed. M. E. Maruish (Mahwah, NJ: Lawrence Erlbaum Associates Publishers), 363–377. 10.1037/t29280-000

[B30] Eckert M. A. (2011). Slowing down: age-related neurobiological predictors of processing speed. Front. Neurosci. 5:25. 10.3389/fnins.2011.00025 21441995 PMC3061488

[B31] Eichenbaum H. (2017). Prefrontal–hippocampal interactions in episodic memory. Nat. Rev. Neurosci. 18, 547–558. 10.1038/nrn.2017.74 28655882

[B32] Faul F. Erdfelder E. Buchner A. Lang A.-G. (2009). Statistical power analyses using G^*^Power 3.1: tests for correlation and regression analyses. Behav. Res. Methods 41, 1149–1160. 10.3758/BRM.41.4.1149 19897823

[B33] Freeman D. Reeve S. Robinson A. Ehlers A. Clark D. M. Spanlang B.. (2017). Virtual reality in the assessment, understanding, and treatment of mental health disorders. Psychol. Med. 47, 2393–2400. 10.1017/S003329171700040X 28325167 PMC5964457

[B34] Gamito P. Oliveira J. Lopes P. Brito R. Morais D. Caçoete C.. (2017). Cognitive training through mHealth for individuals with substance use disorder. Methods Inf. Med. 56, 156–161. 10.3414/ME16-02-0012 28116414

[B35] Gamito P. Oliveira J. Lopes P. Brito R. Morais D. Silva D.. (2014). Executive functioning in alcoholics following an mHealth cognitive stimulation program: randomized controlled trial. J. Med. Internet Res. 16:e102. 10.2196/jmir.2923 24742381 PMC4019779

[B36] Gamito P. Oliveira J. Matias M. Cunha E. Brito R. Lopes P. F.. (2021). Virtual reality cognitive training among individuals with alcohol use disorder undergoing residential treatment: pilot randomized controlled trial. J. Med. Internet Res. 23, 1–10. 10.2196/18482 33512329 PMC7880813

[B37] Giancola P. Tarter R. (1999). Executive cognitive functioning and risk for substance abuse. Psychol. Sci. 10, 203–205. 10.1111/1467-9280.00135

[B38] Grant D. A. Berg E. A. (1948). A behavioral analysis of degree of reinforcement and ease of shifting to new responses in a Weigl-type card-sorting problem. J. Exp. Psychol. 38, 404–411. 10.1037/h0059831 18874598

[B39] Hagen E. Erga A. Hagen K. Nesvåg S. McKay J. Lundervold A.. (2016). Assessment of executive function in patients with substance use disorder: a comparison of inventory- and performance-based assessment. J. Subst. Abuse Treat. 66, 1–8. 10.1016/j.jsat.2016.02.010 27211990

[B40] Hanson K. L. Medina K. L. Padula C. B. Tapert S. F. Brown S. A. (2011). Impact of adolescent alcohol and drug use on neuropsychological functioning in young adulthood: 10-year outcomes. J. Child Adolescent Substance Abuse 20, 135–154. 10.1080/1067828X.2011.555272 21532924 PMC3083020

[B41] Hildebrand M. (2015). The psychometric properties of the Drug Use Disorders Identification Test (DUDIT): a review of recent research. J. Substance Abuse Treat. 53, 52–59. 10.1016/j.jsat.2015.01.008 25682718

[B42] Hill-Bowen L. D. Riedel M. C. Salo T. Flannery J. S. Poudel R. Laird A. R.. (2022). Convergent gray matter alterations across drugs of abuse and network-level implications: a meta-analysis of structural MRI studies. Drug Alcohol Depend. 240:109625. 10.1016/j.drugalcdep.2022.109625 36115222

[B43] Höijer I. Ilonen T. Löyttyniemi E. Salokangas R. K. R. (2020). Neuropsychological performance in patients with substance use disorder with and without mood disorders. Nord. J. Psychiatry 74, 444–452. 10.1080/08039488.2020.1734079 32134345

[B44] Hopewell S. Chan A.-W. Collins G. S. Hróbjartsson A. Moher D. Schulz K. F.. (2025). CONSORT 2025 statement: updated guideline for reporting randomised trials. Lancet 405:10489. 10.1016/S0140-6736(25)00672-5 40245901

[B45] Hung Y. Gaillard S. Yarmak P. Arsalidou M. (2018). Dissociations of cognitive inhibition, response inhibition, and emotional interference: voxelwise ALE meta-analyses of fMRI studies. Hum. Brain Mapp. 39, 4065–4082. 10.1002/hbm.24232 29923271 PMC6866358

[B46] IBM Corp (2022). IBM SPSS Statistics for Mac (29.0). Armonk, NY: IBM Corp.

[B47] Joseph E. Chandrasekaran S. Uma T. Easvaradoss V. (2017). Improving perceptual reasoning in school children through chess training. World Acad. Sci. Eng. Technol. Int. J. Psychol. Behav. Sci. 4, 1122–1125. Available online at: https://escholarship.org/uc/item/6qr3751h (Accessed May 15, 2025).

[B48] Khemiri L. Brynte C. Stunkel A. Klingberg T. Jayaram-Lindström N. (2019). Working memory training in alcohol use disorder: a randomized controlled trial. Alcoholism Clin. Exp. Res. 43, 135–146. 10.1111/acer.13910 30462837 PMC6587824

[B49] Kim T. Kim S. Kang J. Kwon M. Lee S.-H. (2022). The common effects of sleep deprivation on human long-term memory and cognitive control processes. Front. Neurosci. 16:883848. 10.3389/fnins.2022.883848 35720688 PMC9201256

[B50] Kloft L. Monds L. A. Blokland A. Ramaekers J. G. Otgaar H. (2021). Hazy memories in the courtroom: a review of alcohol and other drug effects on false memory and suggestibility. Neurosci. Biobehav. Rev. 124, 291–307. 10.1016/j.neubiorev.2021.02.012 33587958

[B51] Kopelman M. D. Wilson B. A. Baddeley A. D. (1997). The Handbook of Memory Disorders. Chichester: John Wiley and Sons.

[B52] Langener S. Van Der Nagel J. van Manen J. Markus W. Dijkstra B. De Fuentes-Merillas L.. (2021). Clinical relevance of immersive virtual reality in the assessment and treatment of addictive disorders: a systematic review and future perspective. J. Clin. Med. 10:3658. 10.3390/jcm10163658 34441953 PMC8396890

[B53] Li W. Li Y. Zhu L. Qin S. (2020). False memory in individuals with substance use disorders: a systematic review. Front. Psychol. 11:540306. 10.3389/fpsyg.2020.540306

[B54] Liu Y. Chen Y. Fraga-González G. Szpak V. Laverman J. Wiers R. W.. (2022). Resting-state EEG, substance use and abstinence after chronic use: a systematic review. Clin. EEG Neurosci. 53, 344–366. 10.1177/15500594221076347 35142589

[B55] Mackey A. P. Hill S. S. Stone S. I. Bunge S. A. (2011). Differential effects of reasoning and speed training in children. Dev. Sci. 14, 582–590. 10.1111/j.1467-7687.2010.01005.x 21477196

[B56] Man D. W. K. (2020). Virtual reality-based cognitive training for drug abusers: a randomised controlled trial. Neuropsychol. Rehabil. 30, 315–332. 10.1080/09602011.2018.1468271 29734923

[B57] Marceau E. M. Berry J. Lunn J. Kelly P. J. Solowij N. (2017). Cognitive remediation improves executive functions, self-regulation and quality of life in residents of a substance use disorder therapeutic community. Drug Alcohol Depend. 178, 150–158. 10.1016/j.drugalcdep.2017.04.023 28651150

[B58] Maxwell S. E. Delaney H. D. (2004). Designing Experiments and Analyzing Data: A Model Comparison Perspective, 2nd Edn. Mahwah, NJ: Lawrence Erlbaum Associates Publishers. 10.4324/9781410609243

[B59] Melugin P. R. Nolan S. O. Siciliano C. A. (2021). Bidirectional causality between addiction and cognitive deficits. Int. Rev. Neurobiol. 157, 371–407. 10.1016/bs.irn.2020.11.001 33648674 PMC8566632

[B60] Miles S. Howlett C. A. Berryman C. Nedeljkovic M. Lorimer Moseley and, G. Phillipou A. (2021). Considerations for using the Wisconsin Card Sorting Test to assess cognitive flexibility. Behav. Res. Methods 53, 2083–2091. 10.3758/s13428-021-01551-3 33754321

[B61] Mueller S. T. Piper B. J. (2014). The psychology experiment building language (PEBL) and PEBL test battery. J. Neurosci. Methods 222, 250–259. 10.1016/j.jneumeth.2013.10.024 24269254 PMC3897935

[B62] Nardo T. Batchelor J. Berry J. Francis H. Jafar D. Borchard T. (2022). Cognitive remediation as an adjunct treatment for substance use disorders: a systematic review. Neuropsychol. Rev. 32, 161–191. 10.1007/s11065-021-09506-3 33871785

[B63] Nguyen L. Murphy K. Andrews G. (2019). Cognitive and neural plasticity in old age: a systematic review of evidence from executive functions cognitive training. Ageing Res. Rev. 53:100912. 10.1016/j.arr.2019.100912 31154013

[B64] Papaioannou T. Voinescu A. Petrini K. Stanton Fraser D. (2022). Efficacy and moderators of virtual reality for cognitive training in people with dementia and mild cognitive impairment: a systematic review and meta-analysis. J. Alzheimer. Dis. 88, 1341–1370. 10.3233/JAD-210672 35811514

[B65] Pardilla-Delgado E. Payne J. D. (2017). The impact of sleep on true and false memory across long delays. Neurobiol. Learn. Mem. 137, 123–133. 10.1016/j.nlm.2016.11.016 27903437

[B66] Parvaz M. Rabin R. Adams F. Goldstein R. (2022). Structural and functional brain recovery in individuals with substance use disorders during abstinence: a review of longitudinal neuroimaging studies. Drug Alcohol Depend. 232:109319. 10.1016/j.drugalcdep.2022.109319 35077955 PMC8885813

[B67] Pazoki Z. Kheirkhah M. T. Gharibzadeh S. (2024). Cognitive training interventions for substance use disorders: what they really offer? Front. Public Health 12:1388935. 10.3389/fpubh.2024.1388935 38694981 PMC11061450

[B68] Peacock A. Leung J. Larney S. Colledge S. Hickman M. Rehm J.. (2018). Global statistics on alcohol, tobacco and illicit drug use: 2017 status report. Addiction 113, 1905–1926. 10.1111/add.14234 29749059

[B69] PEBL Project (2018). PEBL Test Battery [Computer Software] (2.0). Available online at: http://pebl.sourceforge.net/ (Accessed May 20, 2025).

[B70] Pennington D. L. Reavis J. V. Cano M. T. Walker E. Batki S. L. (2022). The Impact of exercise and virtual reality executive function training on cognition among heavy drinking veterans with traumatic brain injury: a pilot feasibility study. Front. Behav. Neurosci. 16:802711. 10.3389/fnbeh.2022.802711 35391785 PMC8981916

[B71] Potvin S. Pelletier J. Grot S. Hébert C. Barr A. M. Lecomte T. (2018). Cognitive deficits in individuals with substance use disorders: a meta-analysis. Addiction 113, 1561–1573. 10.1111/add.14146 29407687

[B72] Potvin S. Stavro K. Rizkallah E. Pelletier J. (2014). Cocaine and cognition: a systematic quantitative review. J. Addiction Med. 8, 368–376. 10.1097/ADM.0000000000000066 25187977

[B73] Radloff L. S. (1977). The CES-D scale: a self-report depression scale for research in the general population. Appl. Psychol. Meas. 1, 385–401. 10.1177/014662167700100306

[B74] Rass O. Schacht R. L. Buckheit K. Johnson M. W. Strain E. C. Mintzer M. Z. (2015). A randomized controlled trial of the effects of working memory training in methadone maintenance patients. Drug Alcohol Depend. 156, 38–46. 10.1016/j.drugalcdep.2015.08.012 26404954 PMC4633307

[B75] Reitan R. M. (1958). Validity of the Trail Making Test as an indicator of organic brain damage. Percept. Motor Skills 8, 271–276. 10.2466/pms.1958.8.3.271

[B76] Reitan R. M. Wolfson D. (1993). The Halstead – Reitan Neuropsychological Test Battery: Theory and Clinical Interpretation, 2nd Edn. Tucson, AZ: Neuropsychology Press. Available online at: https://search.library.wisc.edu/catalog/9910209689302121

[B77] Rezapour T. Hatami J. Farhoudian A. Sofuoglu M. Noroozi A. Daneshmand R.. (2019). Cognitive rehabilitation for individuals with opioid use disorder: a randomized controlled trial. Neuropsychol. Rehabil. 29, 1273–1289. 10.1080/09602011.2017.1391103 29161998

[B78] Roberts C. Horton A. M. J. (2001). Using the trail making test to screen for cognitive impairment in a drug abuse treatment sample. Int. J. Neurosci. 109, 273–280. 10.3109/00207450108986538 11699333

[B79] Roediger H. L. McDermott K. B. (1995). Creating false memories: remembering words not presented in lists. J. Exp. Psychol. Learn. Memory Cogn. 21, 803–814. 10.1037//0278-7393.21.4.803

[B80] Rolland B. D'Hondt F. Montègue S. Brion M. Peyron E. D'Aviau de Ternay J.. (2019). A patient-tailored evidence-based approach for developing early neuropsychological training programs in addiction settings. Neuropsychol. Rev. 29, 103–115. 10.1007/s11065-018-9395-3 30607658

[B81] Rubo M. Messerli N. Munsch S. (2021). The human source memory system struggles to distinguish virtual reality and reality. Comput. Hum. Behav. Rep. 4:100111. 10.1016/j.chbr.2021.100111

[B82] Rupp C. I. Kemmler G. Kurz M. Hinterhuber H. Wolfgang Fleischhacker W. (2012). Cognitive remediation therapy during treatment for alcohol dependence. J. Stud. Alcohol Drugs 73, 625–634. 10.15288/jsad.2012.73.625 22630801

[B83] Schmiedek F. Lövdén M. Lindenberger U. (2014). Younger adults show long-term effects of cognitive training on broad cognitive abilities over 2 years. Dev. Psychol. 50, 2304–2310. 10.1037/a0037388 25019946

[B84] Serra M. La Corte V. Migliaccio R. Brazzarola M. Zannoni I. Pradat-Diehl P.. (2014). Confabulators mistake multiplicity for uniqueness. Cortex 58, 239–247. 10.1016/j.cortex.2014.06.011 25080079

[B85] Shao X. Chen C. Loftus E. F. Xue G. Zhu B. (2022). Dynamic changes in neural representations underlie the repetition effect on false memory. Neuroimage 259:119442. 10.1016/j.neuroimage.2022.119442 35788042

[B86] Shen J. Hua G. Li C. Liu S. Liu L. Jiao J. (2023). Prevalence, incidence, deaths, and disability-adjusted life-years of drug use disorders for 204 countries and territories during the past 30 years. Asian J. Psychiatr. 86:103677. 10.1016/j.ajp.2023.103677 37348194

[B87] Sofuoglu M. DeVito E. E. Waters A. J. Carroll K. M. (2013). Cognitive enhancement as a treatment for drug addictions. Neuropharmacology 64, 452–463. 10.1016/j.neuropharm.2012.06.021 22735770 PMC3445733

[B88] Steingass H. P. Marsh F. N. Gordon B. (1994). Neuropsychological dysfunction in detoxified alcoholics: return to normality over 10 years? Alcohol. Clin. Exp. Res. 18, 302–308. 10.1111/j.1530-0277.1994.tb00019.x 8048732

[B89] Stroop J. R. (1935). Studies of interference in serial verbal reactions. J. Exp. Psychol. 18, 643–662. 10.1037/h0054651

[B90] Sullivan A. Ridley N. Monds L. A. Logge W. Hurzeler T. Morley K. C. (2023). Assessing the validity of the Wechsler Adult Intelligence Scale (WAIS-IV) in predicting completion in a long-term residential rehabilitation for substance use problems. Appl. Neuropsychol. Adult 30, 561–566. 10.1080/23279095.2021.1967954 34455866

[B91] Tiego J. Testa R. Bellgrove M. A. Pantelis C. Whittle S. (2018). A hierarchical model of inhibitory control. Front. Psychol. 9:1339. 10.3389/fpsyg.2018.01339 30123154 PMC6085548

[B92] Towe S. L. Hartsock J. T. Xu Y. Meade C. S. (2021). Web-based cognitive training to improve working memory in persons with co-occurring HIV infection and cocaine use disorder: outcomes from a randomized controlled trial. AIDS Behav. 25, 1542–1551. 10.1007/s10461-020-02993-0 32749625 PMC7858692

[B93] Traut H. J. Guild R. M. Munakata Y. (2021). Why does cognitive training yield inconsistent benefits? A meta-analysis of individual differences in baseline cognitive abilities and training outcomes. Front. Psychol. 12:662139. 10.3389/fpsyg.2021.662139 34122249 PMC8187947

[B94] Tröndle M. Langer N. (2024). Decomposing neurophysiological underpinnings of age-related decline in visual working memory. Neurobiol. Aging 139, 30–43. 10.1016/j.neurobiolaging.2024.03.004 38593526

[B95] Tsamitros N. Sebold M. Gutwinski S. Beck A. (2021). Virtual reality-based treatment approaches in the field of substance use disorders. Curr. Addiction Rep. 8, 399–407. 10.1007/s40429-021-00377-5

[B96] Van Dam N. T. Earleywine M. (2011). Validation of the center for epidemiologic studies depression scale - revised (CESD-R): pragmatic depression assessment in the general population. Psychiatry Res. 186, 128–132. 10.1016/j.psychres.2010.08.018 20843557

[B97] Van Der Plas E. Crone E. Van Den Wildenberg W. Tranel D. Bechara A. (2009). Executive control deficits in substance-dependent individuals: a comparison of alcohol, cocaine, and methamphetamine and of men and women. J. Clin. Exp. Neuropsychol. 31, 706–719. 10.1080/13803390802484797 19037812 PMC2829119

[B98] Verdejo-Garcia A. Garcia-Fernandez G. Dom G. (2019). Cognition and addiction. Dial. Clin. Neurosci. 21, 281–290. 10.31887/DCNS.2019.21.3/gdom 31749652 PMC6829168

[B99] Wankhede N. L. Koppula S. Ballal S. Doshi H. Kumawat R. Raju S.s.. (2025). Virtual reality modulating dynamics of neuroplasticity: innovations in neuro-motor rehabilitation. Neuroscience 566, 97–111. 10.1016/j.neuroscience.2024.12.040 39722287

[B100] Wechsler D. (2008). Wechsler Adult Intelligence Scale – Fourth Edition Administration and Scoring Manual. San Antonio, TX: Pearson. 10.1037/t15169-000

[B101] Wechsler D. (2009). Wechsler Memory Scale – Fourth Edition (WMS-IV) Technical and Interpretive Manual. San Antonio, TX: Pearson.

[B102] Willis S. L. Schaie K. W. (1986). Training the elderly on the ability factors of spatial orientation and inductive reasoning. Psychol. Aging 1, 239–247. 10.1037/0882-7974.1.3.239 3267404

[B103] Wu J. Ma Y. Ren Z. (2020). Rehabilitative effects of virtual reality technology for mild cognitive impairment: a systematic review with meta-analysis. Front. Psychol. 11:1811. 10.3389/fpsyg.2020.01811 33101098 PMC7545425

[B104] Yan H. Xiao S. Fu S. Gong J. Qi Z. Chen G.. (2023). Functional and structural brain abnormalities in substance use disorder: a multimodal meta-analysis of neuroimaging studies. Acta Psychiatr. Scand. 147, 345–359. 10.1111/acps.13539 36807120

[B105] Zhong D. Chen L. Feng Y. Song R. Huang L. Liu J.. (2021). Effects of virtual reality cognitive training in individuals with mild cognitive impairment: a systematic review and meta-analysis. Int. J. Geriatric Psychiatry 36, 1829–1847. 10.1002/gps.5603 34318524

[B106] Zuo L. (2022). “Comparison between the traditional and computerized cognitive training programes in treating mild cognitive impairment,” in 2022 2nd International Conference on Electronic Information Engineering and Computer Technology (EIECT) (Piscataway, NJ: IEEE), 119–124. 10.1109/EIECT58010.2022.00029

